# System Design Based on Biological Olfaction for Meat Analysis Using E-Nose Sensors

**DOI:** 10.1021/acsomega.4c04791

**Published:** 2024-07-17

**Authors:** Ozgun Boray Yurdakos, Ozge Cihanbegendi

**Affiliations:** † Basic Oncology Department, Ege University, 35100 Izmir, Turkiye; ‡ Department of Electrical and Electronics Engineering, Dokuz Eylul University, 35210 Izmır, Turkiye

## Abstract

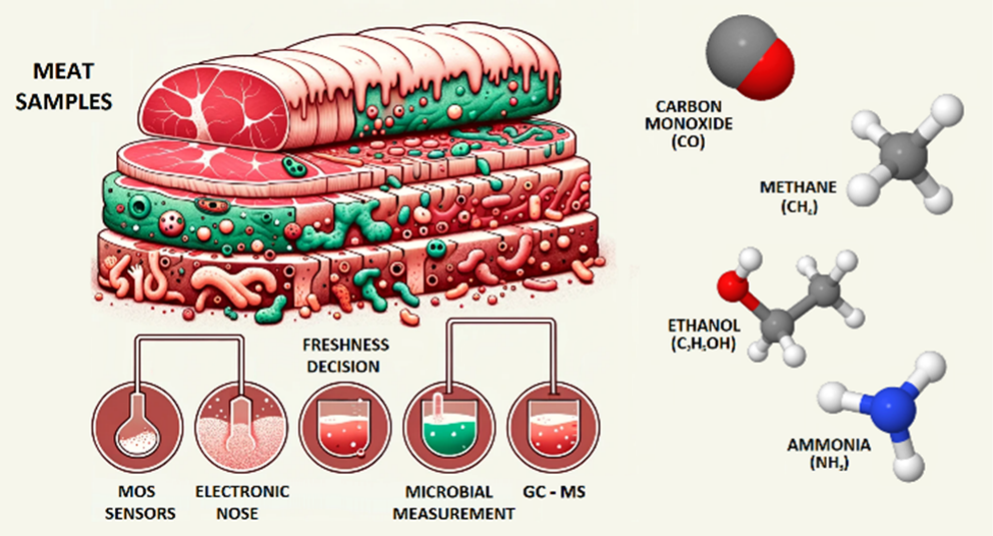

The deterioration of food, especially in meat products, can lead to serious health problems. Even with modern preservation technologies, a significant amount of food is lost due to microbial deterioration. As the very first step of the preservation process, the microflora that grows during the storage time and in spoiling foods should be well-known to identify critical levels. Electronic nose and gas chromatography analysis systems can provide sensitive and promising results. Similarly, bacterial analysis is an important process for determining bacterial groups that result in the emergence of such gases in gas chromatography-mass spectrometry (GC-MS) analysis during the degradation time. This study aims to determine the degradation levels for some meat types under different environmental conditions, such as temperature and duration, to compare with other measurement techniques for evaluating the verification of data. E-nose device, developed in this study, can detect carbon monoxide (CO), methane (CH_4_), ethanol (C_2_H_5_OH), and ammonia (NH_3_) using metal oxide semiconductor (MOS) sensors. In order to test sensory measurements during this period, GC-MS and microbial measurements were used. E-nose measurements show that the results are in accord with each other. This system can easily be made portable, occupying very little space.

## 1 Introduction

An electronic nose, often abbreviated as “e-nose”, is an analytical device designed to mimic the human olfactory system’s ability to detect and identify odors and volatile compounds. It includes an array of sensors capable of detecting and analyzing the complex mixture of volatile organic compounds (VOCs) present in the air or gases, along with signal processing and pattern recognition units.^1^ Commercial electronic noses find applications in diverse fields, including environmental monitoring, medical instrumentation and healthcare, food, chemical, automotive industries, agriculture, pharmaceuticals, mining, security and safety, cosmetics, and perfume industry. They are employed for odor recognition, detection of harmful chemicals, and analysis of various food products, enhancing quality inspection, safety, and efficiency in various industries and sectors.^2,3^

Using diverse chemical gas sensors and appropriate statistical approaches, we enable the identification of complex odors. In the evaluation of volatile compounds in food, cosmetics, and other daily life, the availability of commercial facilities has resulted in a significant increase in research on the application of electronic noses. Piezoelectric crystals, organic conductive polymers, metal oxide semiconductor field effect transistors (MOSFETs), metal oxide semiconductor (MOS) sensors, surface acoustic wave (SAW) sensors, optical sensors, and quartz crystal microbalance (QCM) sensors are among the elements commonly found in the list of industrial gas sensors.^2,4−6^ The process of recognizing an odor begins with obtaining the responses of each sensor. Gas sensors, including MOS sensors commonly used as part of electronic noses, generate signals through both chemical reactions and physical adsorption processes, known as physisorption. These sensors typically operate by detecting alterations in their electrical properties, such as resistance, capacitance, charge carrier mobility, or threshold value. These changes, induced by the adsorption of gas molecules onto the sensor’s surface, are used to ascertain the presence and concentration of the target gas.^2,6^ In recent years, MOS sensors have become the primary choice for designing highly sensitive, stable, and low-cost gas sensors for real-life applications, thanks to their inherent physical and chemical properties.^7^ Additionally, organic field effect transistors (OFETs) offer the opportunity for ultrahigh sensitivity in gas sensor applications.^8−10^

Analysis that has many variables such as radial basic functions, artificial neural network, and simple graphical evaluation can be named statistical analysis techniques. Different analytical methods in food quality assessment exist based on passive sampling, chromatography, spectroscopy, and sensor.^11,12^ Applications of electronic noses in the food industry encompass freshness determination, process monitoring, quality control, authenticity analysis, and shelf life estimation.^13^ Beyond its role in assessing quality and flavor in the food industry, the determination of meat freshness under varying conditions using electronic nose sensor arrays holds greater significance for health. Several research studies have already been conducted on fish, cheese, mushrooms, sugar, apple, coffee, and other beverages.^14,15^ Another e-nose study was focused on meat, rice, and bread degradation levels. Chen et al.^16^ built a lab-made electronic sensor array to detect the freshness level of various red meats over a 7 day period and then corrected these sensor outputs using human sensory evaluations. Although the temperature values and the number of categories of meat freshness are different, their approach to determining the freshness levels of meats was quite similar to this study. Aside from meats, similarly, Gull et al.^17^ used MQ series sensors^18^ to detect volatile gases such as CH_4_, CO_2_, NH_3_, etc. They also collected data from cooked samples.^16^ Their correction method was based on a machine learning system. However, the focus of the study was not evaluating the change in values by collecting the e-nose data over time.

There are also other studies using an e-nose system that aim to detect the adulteration of pork in minced red meat or the quality of meat, respectively.^19,20^ Furthermore, a wireless detection method has recently been proposed.^21^

In designing these systems, it is crucial to ensure a high level of repeatability and stability, especially for long-term use. This involves maintaining the ability to consistently analyze samples on the same sensor array throughout the measurement period and ensuring that various sensor groups and tools can reproduce identical sample patterns. While electronic nose sensors provide valuable tools for food analysis, they do come with challenges such as calibration, sensor drift, cross-sensitivity, and susceptibility to environmental interference. Therefore, careful consideration of sample preparation is essential when implementing e-nose systems for food quality and safety assessment. Addressing these challenges is imperative to guarantee accurate and reliable results.^2^

Similar to the case of olfactory systems, each cell within a sensor array functions as an individual receptor, responding to various odorants with varying intensities. These distinctions are transformed into electrical signals through preprocessing and are subsequently characterized by the model recognition system. This response from the array is formed as an electronic nose to allow for a specific odor in each odor group.^11,22^

Over the past 100 years, many techniques have been applied in several scientific fields to detect aroma-active compounds in food. Among the various techniques, gas chromatography and mass spectrometry (GC-MS) is an effective and commonly used technique for aroma analysis such as the odor of foods.^23^ GC-MS is used as a conventional method for odor detection systems due to the intensive use of chemical analysis methods. This conventional approach enabled the modeling of such a system called E-nose with rapid advances in smell sensing technologies in the literature. GC-MS technique is widely accepted for its high accuracy in odor detection and food analysis, enabling the detection and monitoring of various parameters of food quality and adulteration.^24^

In the following sections, there will be experimental layouts including various steps of the study. The purpose of this study was to identify the degradation levels of a punch of minced beef meat and flaked poultry meat at intervals of 1–7 days under different environmental conditions such as temperatures of 4 and 22 °C using a sensor array. Additionally, GC-MS and microbiological analyses were performed at the beginning and end of the study to validate the sensory outputs during this time period. The sensory outputs during this period were verified at the start and end phases of the study. The reason for choosing these temperature values is to represent the refrigerator temperature by 4 °C and the mean outside temperature by 22 °C. These two numbers, which will be included in the final data, will be useful in testing the everyday conditions of meat consumption. The lack of such designs in the literature, including dual measurement of meats by the same sensor array as with chemical approaches, provided strong motivation to carry out this method. With this technique, it is noticed that one compound for each meat type can be decisive in terms of the detection of the spoilage. Moreover, as being portable and simple, this device can be used in supermarkets, food stores, and restaurants to check the meat freshness.

## 2 Materials and Methods

**Figure 1:**
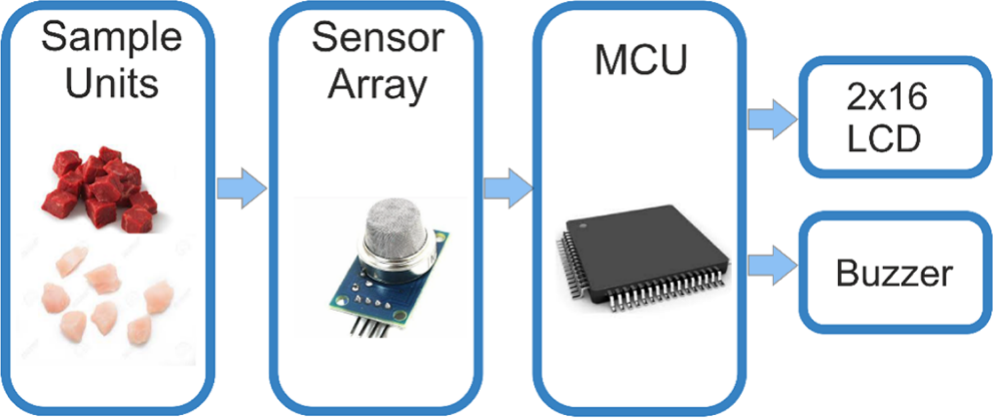
Block diagram of the system.

The study includes a few procedures depicted in Figure 1 that demonstrate the principle of the e-nose system created based on sensor array outputs to determine sample freshness. In the beginning, samples were transported close enough to the sensor array using an injector received from the chamber to detect what type of gas content existed. This mixture of gases was separated according to the sensor type for each sensing among all of the gas contents. This value was then calculated over analog outputs to ascertain whether or not the critical value was surpassed in terms of determined values for each one previously. According to this exceeded limit, the system gave a deterioration warning number of 1–4. If such a value is not observed over the sample, then logic 0 is produced to carry on measurements.

**Figure 2:**
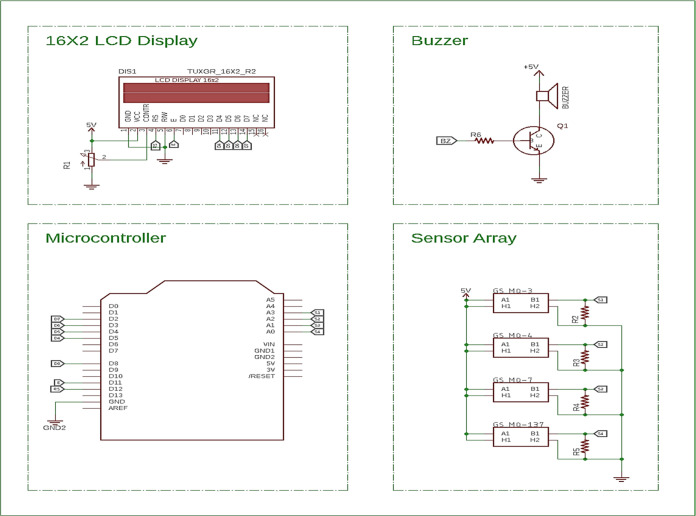
Detailed circuit schema of the e-nose system.

Designing an e-nose system requires many tools. In this study, there are several modules such as the sampling unit, a sensor array, a microcontroller, and liquid-crystal display (LCD) in addition to the buzzer, which can be seen with the connections in detail in Figure 2.

The sampling units where the samples are located serve as the input of the sensor array module. In this transition of analog data from the source to the sensory inputs, the efficiency of the related transfer is not 100% natural. The largest level of loss occurs when gas forms are used as the storage element. Nevertheless, adequate effort is shown to transfer these data into the next module. The sensor array is another analog data part of the design, which senses and sends the data into MCU. In this module, analog data is converted into digital data in order to process or work the outputs that can only work with digital inputs. Finally, the decision (logic 1 or 0) is shown in these outputs. As the elements of MQ series of HANWEI company, MQ7 is used to detect carbon monoxide (CO), MQ4 is used to detect methane (CH_4_), MQ3 is used to identify ethanol (C_2_H_5_OH), and MQ137 is used to detect ammonia (NH_3_) for samples over a 7 day period.^25^ E-nose measurements were made under conditions where room temperature is 22 °C and humidity is %65.

**Figure 3:**
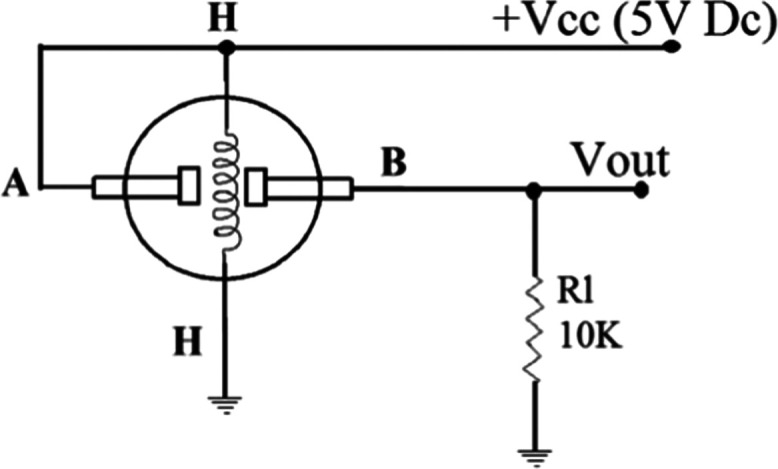
General circuit model of MQ series sensors.^26^

The circuit model of the MQ series sensors is given in Figure 3. Decreasing of the resistance value (*R*_s_) increases the leaking current under a constant direct current (DC) voltage, 5 V. This increased current between A and B allows a higher voltage to be stored on constant load, typically 10 k Ω. Lastly, heater pins, represented as H, provide a stable working temperature during the sensing performance. To summarize, the lowest level to which *R*_s_ can decrease determines the limit to which the gas concentration in the environment can be detected.

**Figure 4:**
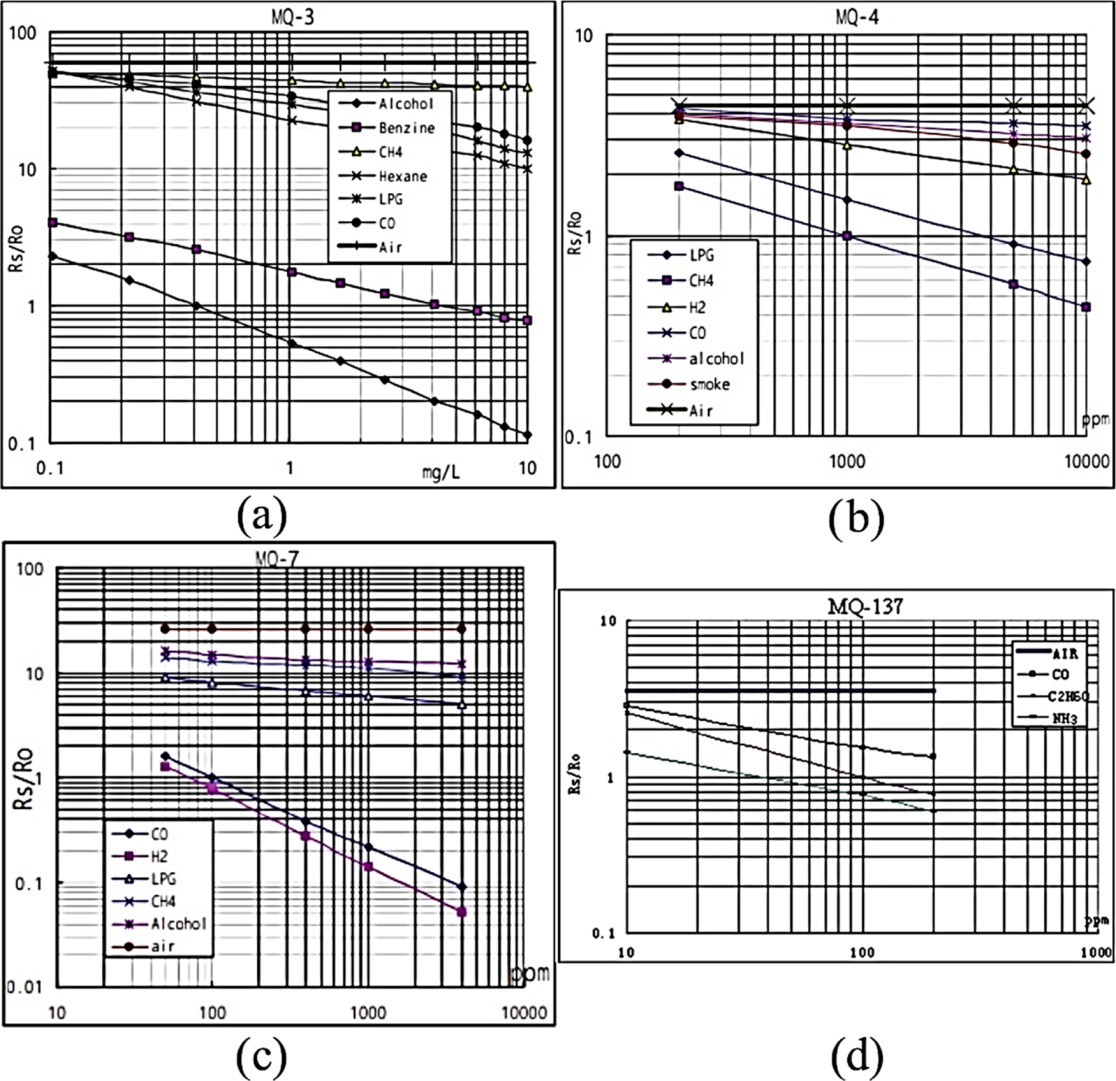
Sensitivity characteristics of sensors, (a) MQ3, for ethanol, (b) MQ4, for methane, (c) MQ7, for carbon monoxide, and (d) MQ137, for ammonia, used in the proposed e-nose design.^18^

In Figure 4, *R*_s_/*R*_o_ characteristics of MQ sensors were shown, where *R*_s_ is the sensor resistance in displayed gases at various concentrations, and *R*_o_ is the sensor resistance in fresh air. MQ series sensors operate inversely proportional to gas concentration in the environment. In other words, as the existence of gas concentration increases, *R*_s_ decreases simultaneously. Consequently, the voltage that is measured at the circuit load via the output pin increases depending on the related gas concentration.

It was determined deliberately to show that there was no direct relation between meat freshness and CO and CH_4_ gases. The reason for selecting these four MOS sensors is mainly related to market availability and being low cost in the market rather than hydrogen sulfide, acetone, dimethyl sulfide, dimethyl ether, or any other VOCs observed in GC-MS. Further, GC-MS measurements were conducted after selecting MOS sensors and obtaining their results in order to verify the sensor measurements.

### 2.1 Microbiological Population Enumeration

The microbial population count is an accurate data source for determining the validity of the gas measurements. If it is possible to predict what type of bacterial growth will be detected prior to the process, then other measurements in terms of emerged gases over the sample will be more useful. As is well-known, this amount of gases is generated almost completely by the respiratory mechanism of such bacteria. The greater the number of bacterial groups, the more reliable the detection of environmental gases. The measurements were carried out in the Microbiology Laboratory at Ege University in Izmir using a device model STOMACHER 400.^27^

#### 2.1.1 Sample Preparation

**Figure 5:**
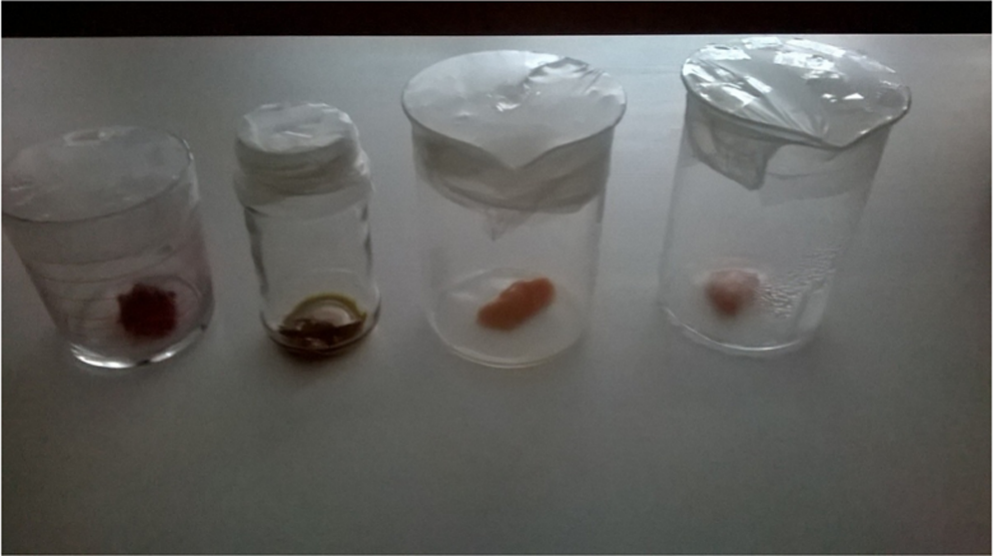
Samples were captured on day 7 after the whole measurements.

Samples were kept in a closed chamber under the same conditions for the duration of the study. Sensory measurements were recorded every single day through an injector over the chamber with neglected gas leakage from one measurement to another. The samples were purchased from a local butcher and were stored at 4 and 22 °C for a period of 7 days. The sample size was set for red meats as minced shapes so that microbiological contamination could be clearly observed. In order to simulate the climatic conditions more realistically, the other sample was kept at its original size. Figure 5 depicts the four samples, of which two of them on the left side are red meat, while the other ones are poultry meat with the same duration at different temperatures (4 and 22 °C). The photograph was taken on the final day of the period before the e-nose measurements.

Fresh minced beef meat and flaked poultry meat provided from a meat shop as 100 g were used for the study. Different meat batches were tested. Meat was transported to the laboratory and held at +4 °C for 1 h.

#### 2.1.2 Microbiological Analysis

First, meat samples (10 g) were transferred to a stomacher bag. Then, the buffer solution was added to the bag, and the mixture was homogenized inside for 60 s. Samples (50 mL) of the appropriate 10-fold serial dilutions were spread on the surface of the appropriate media in Petri dishes and incubated at 25 °C for 48 h. After the colonies were incubated, they were counted.

#### 2.1.3 Scaled Calculation

An easier and more accurate method to determine the microbial count is the plate method, where a food sample is placed on a culture medium plate. After an appropriate incubation period, the number of colonies have formed on the culture medium plate. The scaled calculation represents the number of colonies in the Petri dish after incubation.

#### 2.1.4 Multiplication

Multiplication represents the total number of colonies on the overall Petri dish surface after incubation.

#### 2.1.5 Exponential Factor

The exponential phase of microbial growth is a pattern of balanced growth where all of the cells divide regularly by binary fission and grow by geometric progression. The cells divide at a constant rate depending on the composition of the growth medium and the conditions of incubation.^28^

#### 2.1.6 Total Counts of Bacteria (Total Microbial Count/Total Bacterial Count)

Colony-forming units (CFU) is expressed as colony-forming units per gram or milliliter, depending on the different types of food.

The difference in CFU values of red and white meat at the beginning of microbial analysis may be due to the physical properties of the meat (fat layer content) and the chemical properties (the amount of water content). The fat layer can protect the meat surface, but since enzymatic and chemical deterioration may occur, the rate of microorganism development increases and their reproduction becomes easier. The amount of water contained in meat also affects the development of molds, yeasts, and bacteria. However, there are differences between the microbial load of both meats due to environmental factors such as slaughtering, chopping processes, and various microbial flora that may come from animal shelters.^28^

Temperature is a crucial parameter for the determination of exponential factor for microbial analysis calculations. It can greatly influence the rate of reaction and enzymatic activities. The temperature of foods influences the growth rate of microorganisms and the rate of spoilage. Room temperature is such a high level for this kind of growth.^29^ Microbial growth is suppressed at +4 °C, which is in refrigerator conditions. Therefore, microbial analyses were carried out under +4 °C temperature conditions to examine both types of meat.

While red meat has a strong connective tissue between muscle tissue and bone, poultry meat does not have this strong connective tissue. Therefore, chicken meat is more susceptible to spoilage than red meat. Accordingly, there are differences in microbial growth rates.^30^

Red meat is also easily spoiled by microorganisms because it contains a lot of nutrients, growth factors, etc. The dominant microflora of the red meat constitutes microorganisms from soil, water, and manure. During slaughter, the external surface of the animal may contaminate the meat by direct contact through the above sources and equipment, personnel, and slaughtering area.^31^

There are many factors affecting the microbial load of the meat. First of all, meat has high water content along with dissolved substances such as glycogen, lactic acids, and amino acids. All of these substances can cause microbial growth, which can lead to early food spoilage. Another crucial factor is the redox potential that has an effect on microbial flora. Tissue respiration where O_2_ is consumed and CO_2_ is produced continues after the animal is slaughtered. Anaerobic bacteria that breathe without oxygen dominate the interior of meat and produce lactic acid. While aerobic flora is dominant on the surface of the meat, anaerobic flora is dominant in the interior of the meat. The bulk of the meat becomes anaerobic except on the surface. Another factor is the degree of acidity, in other words, pH value. Acidic pH conditions are not suitable for microbial growth. The pH for meat is in the range of 5.6–7.4. The acidity level of meat varies, depending on the amount of lactic acid, which is the product of the glycolysis reaction that takes place in the muscles after slaughter. The more acid produced, the lower the pH. The degree of acidity depends on the amount of glycogen in the muscle.^32^

### 2.2 Gas Chromatography Topology

Gas chromatography/mass spectrometry is a device that combines gas chromatography and mass spectrometry units to perform structure analysis and quantity detection. The device can be used as a standalone gas chromatography unit or as part of a gas chromatography/mass spectrometry unit. For the identification of compounds separated in a gas chromatography column, gas chromatography/mass spectrometry is widely utilized. Mass spectrometry serves as a detector in gas chromatography/mass spectrometry operations. The chromatogram of the compounds that are sent to mass spectrometry after leaving the chromatographic columns may be obtained and qualitative assessments can be done more accurately by obtaining the mass spectrum of each compound. The compounds are passed through the GC-MS column and sent to the ion source, where they are broken from their weak bonds and turned into smaller compounds, and the spectra of these compounds are calculated. This spectrum is then compared with the spectrum information in the library of the compounds stored in the device, and if it matches above a certain percentage value (>∼95%), it shows that the compound is detected in the sample. The device’s primary advantages are its great separation power, quantitative and quality analysis abilities, and a high level of sensitivity.

All chromatographic data were collected by a Shimadzu QP-2020^33^ gas chromatography-mass spectrometry device, equipped with an RTX-624 VOC column (60 m × 250 μm and film thickness 0.25 μm). As a carrier gas was used, helium (99.999%) was used in gas chromatography. The injection port was set at 250 °C, and all injections were performed with a split ratio of 1:50. For the best separation of compounds, the temperature gradient was applied as follows: 50 °C for 4 min, increased to 250 °C at a rate of 25 °C/min. In this GC method, the total run time is 30 min. The compounds separated in the column were ionized with a fragmentation energy of 70 eV with the ion source set at 230 °C.

## 3 Results

### 3.1 Bacterial Analysis

Bacterial analysis is an essential technique for assessing the gases that emerged during the operation. The counts are made in the beginning of the degradation as *t*_0_ and *t*_1_ in order to determine a reference level of bacterial growth for the minced beef meat and flaked poultry meat.

The total number of aerobic mesophilic bacteria was analyzed in the microbiology laboratory. The aim of this experiment is to determine the 7 day change (*t*_0_ and *t*_1_) in the total number of microorganisms in poultry meat and red meat. For this purpose, a total aerobic mesophilic bacterial count test was performed on days on which samples were purchased (*t*_0_) and after 7 days (*t*_1_). Plate count agar (PCA, MERC) was used in the experiment. The incubation temperature was 30 °C, and the incubation time was 24–48 h. Results and other information on the total number of aerobic mesophilic bacteria analysis are presented in Tables 1 and 2 for poultry meat and red meat. The total number of microorganism in poultry meat was calculated to be 4.4 × 10^4^ cfu/g at *t*_0_ and 7.2 × 10^6^ cfu/g at *t*_1_. The total amount of live bacteria in red meat was calculated to be 6.3 × 10^5^ cfu/g at *t*_0_ and 3.7 × 10^7^ cfu/g at *t*_1_.

**Table 1:** Total Number of Bacteria Living or Occurring in the Samples at 4 °C for *t*_0_

sample type	scaled calculation	multiplication	exponential factor	total counts of bacteria
poultry meat	220 × 2	440	(−2)	4.4 × 10^4^ cfu/g
red meat	31 × 2	63	(−4)	6.3 × 10^5^ cfu/g

**Table 2:** Total Number of Bacteria Living or Occurring in the Samples at 4 °C for *t*_1_

sample type	scaled calculation	multiplication	exponential factor	total counts of bacteria
poultry meat	220 × 2	72	(−5)	7.2 × 10^6^ cfu/g
red meat	185 × 2	370	(−5)	3.7 × 10^7^ cfu/g

### 3.2 GC-MS Analysis

**Figure 6:**
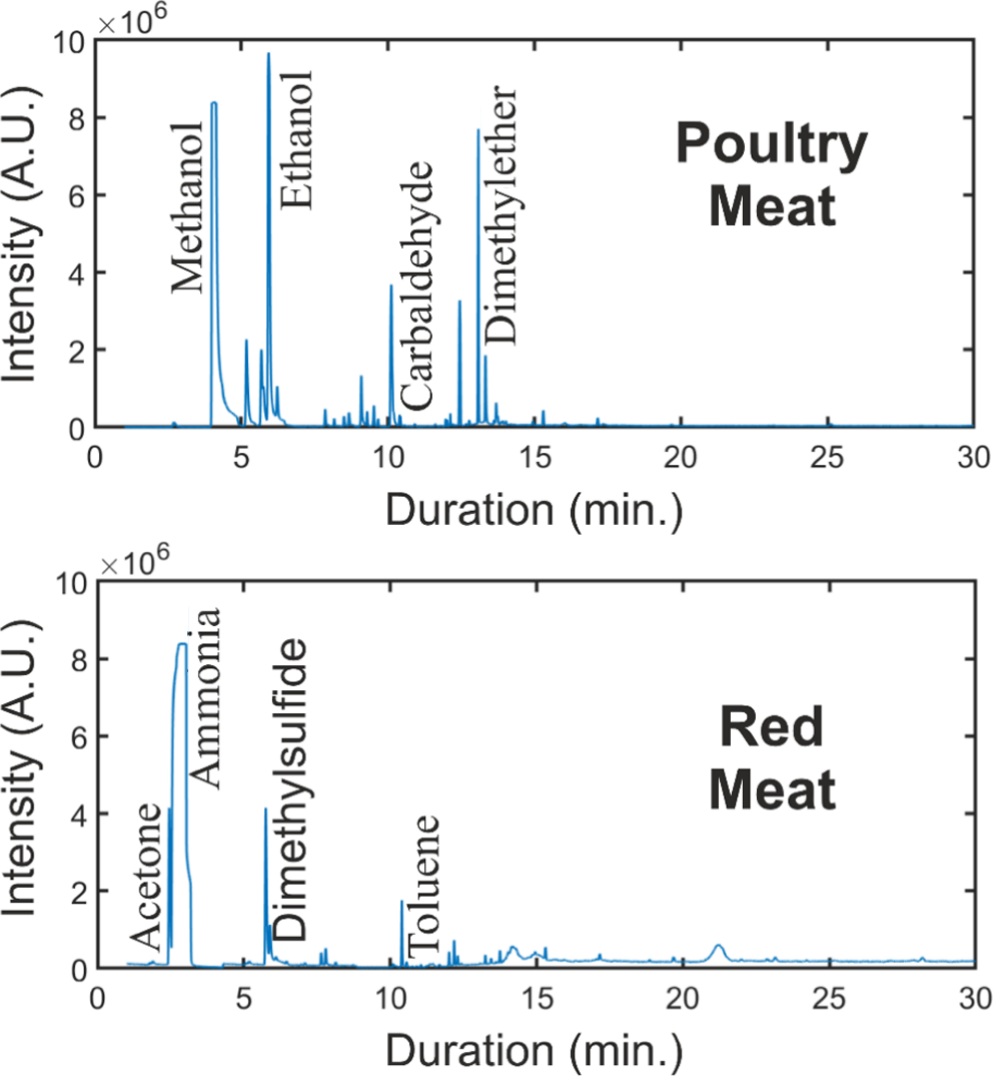
Spectrum view of GC-MS for the sample compounds of poultry and red meat.

As mentioned in the previous section, GC-MS detects substances in liquid or gas phase over the samples. In our laboratory, gas-phase GC-MS is used. As a result, the samples must emit such gases or contain such gases. The GC-MS results show that the expected gas-phase peaks are obtained in both samples, as seen in the figures, and that the whole spectrum of a piece of flaked poultry meat containing the other compounds and minced beef meat including others is obtained. Figure 6 depicts the GC-MS analysis result of the minced red meat sample at 4 °C, with the molecular mass range arranged according to the desired compound in question.

The same analysis was conducted for the poultry meat sample, as well. The total molecular mass spectrum is shown in the same figure. The desired compounds were detected as ammonia and ethanol besides the other compounds methanol, carbaldehyde, and dimethyl ether compounds for the poultry meat and acetone, dimethyl sulfide, and toluene compounds for the red meat, respectively.

### 3.3 E-Nose Analysis

**Figure 7:**
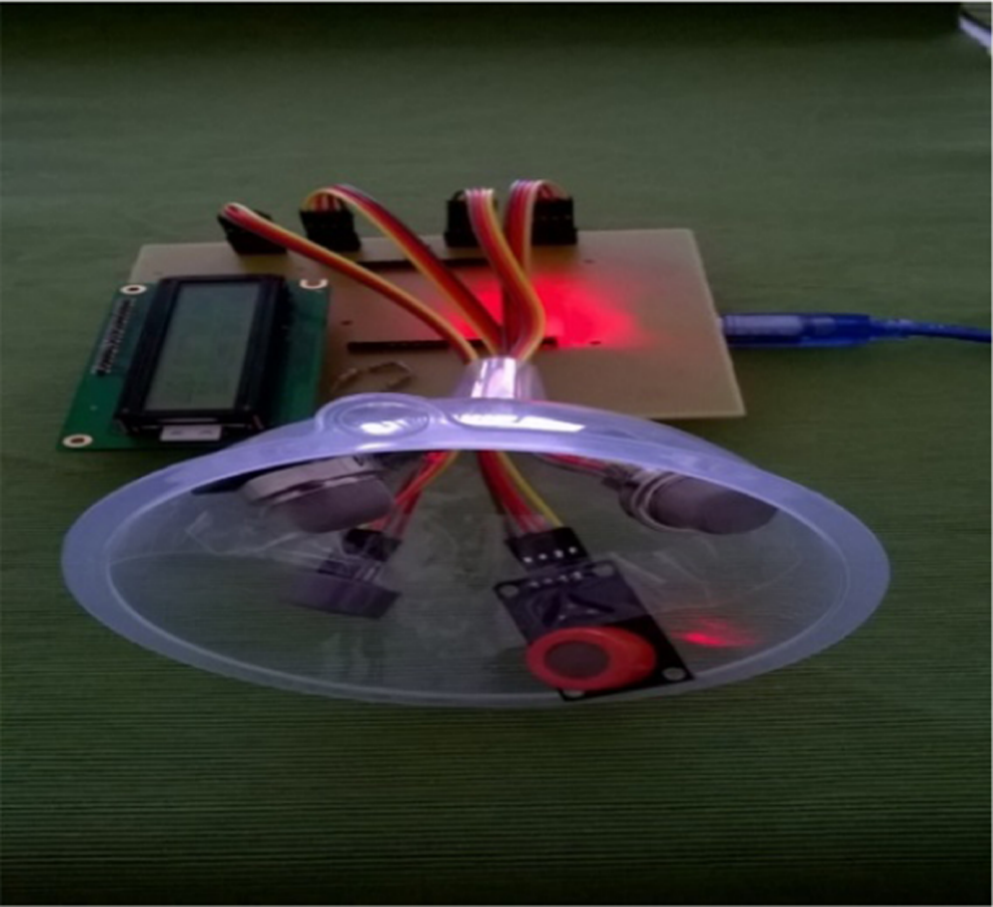
E-nose circuit with the sensor array and LCD unit with a processor connection.

**Figure 8:**
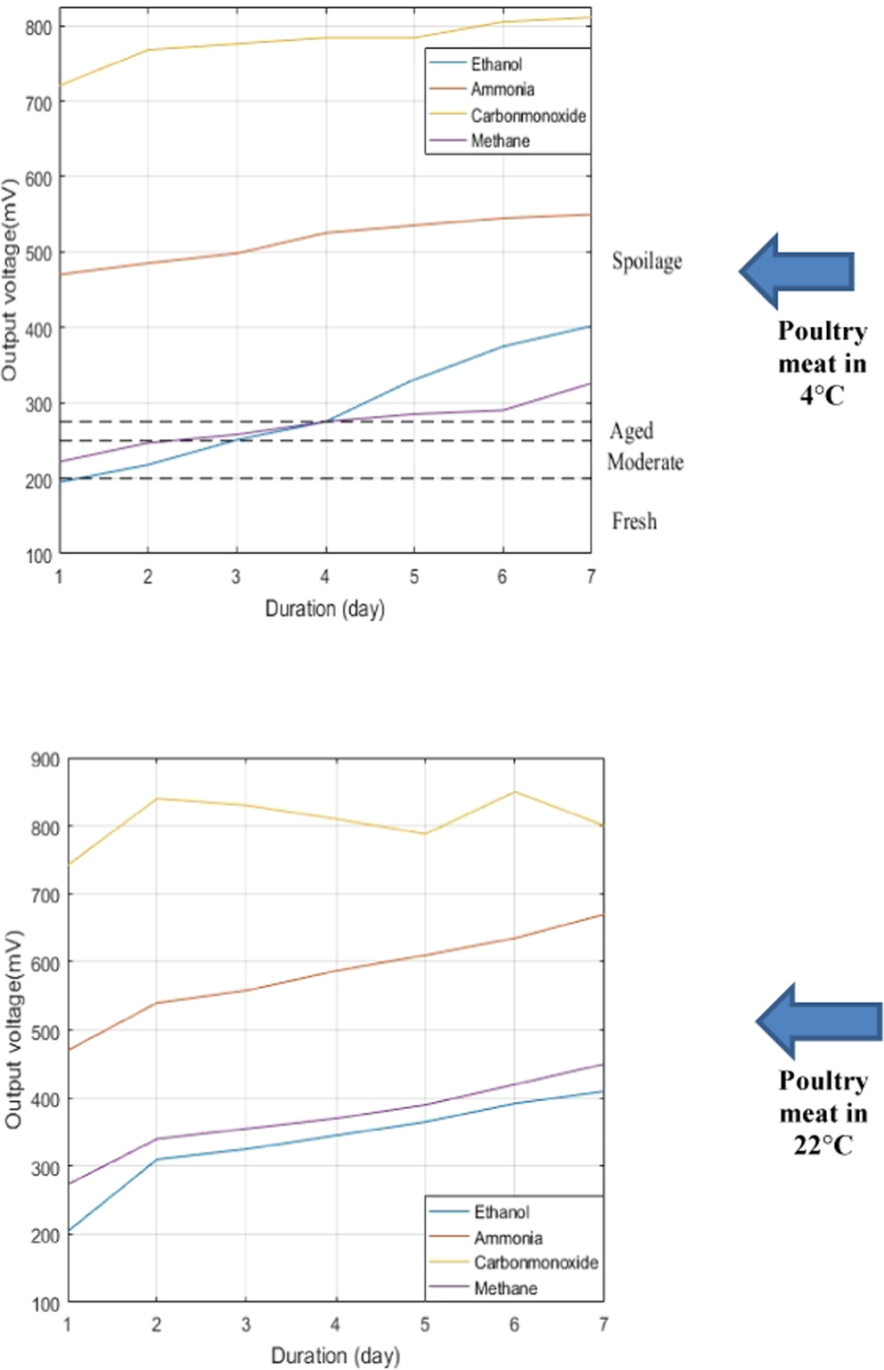
Concentration of ethanol, ammonia, carbon monoxide, and methane gases for poultry meat at (a) 4 °C and (b) 22 °C.

The sensory results were obtained under varying temperatures and durations. Each result that emerged on the output pins of the sensor array is shown in Figures 7 and 8. Rather than studying each sensor output response in relation to the released gas of the samples, as specified in the study, the overall approach is chosen in terms of evaluating a broad perspective. The circuit design is shown in Figure 7 including a parameter sensor array and an LCD unit with a processor connection.

Human observation is used to detect the deterioration levels of poultry meat during the detection process. The degradation levels were determined as 200, 250, and 275 mV depending on the output of the ethanol sensor seen in Figure 8. Under 200 mV, poultry meat is considered fresh up to 1 day after purchase. The smell of poultry meat was very poor, above 275 mV. As a result, it was assumed that spoiling would occur after this threshold.

According to Rajamäki et al.,^34^ the features of curves belonging to sensor array outputs exhibited a comparable spread over time. In comparison to the others, the ethanol sensor response has the greatest time spread. This is an important aspect in determining the reference value to assess sample freshness if it is supported by the percentage change. Among these substances, ethanol had the highest growth rate of more than 100%.

**Figure 9:**
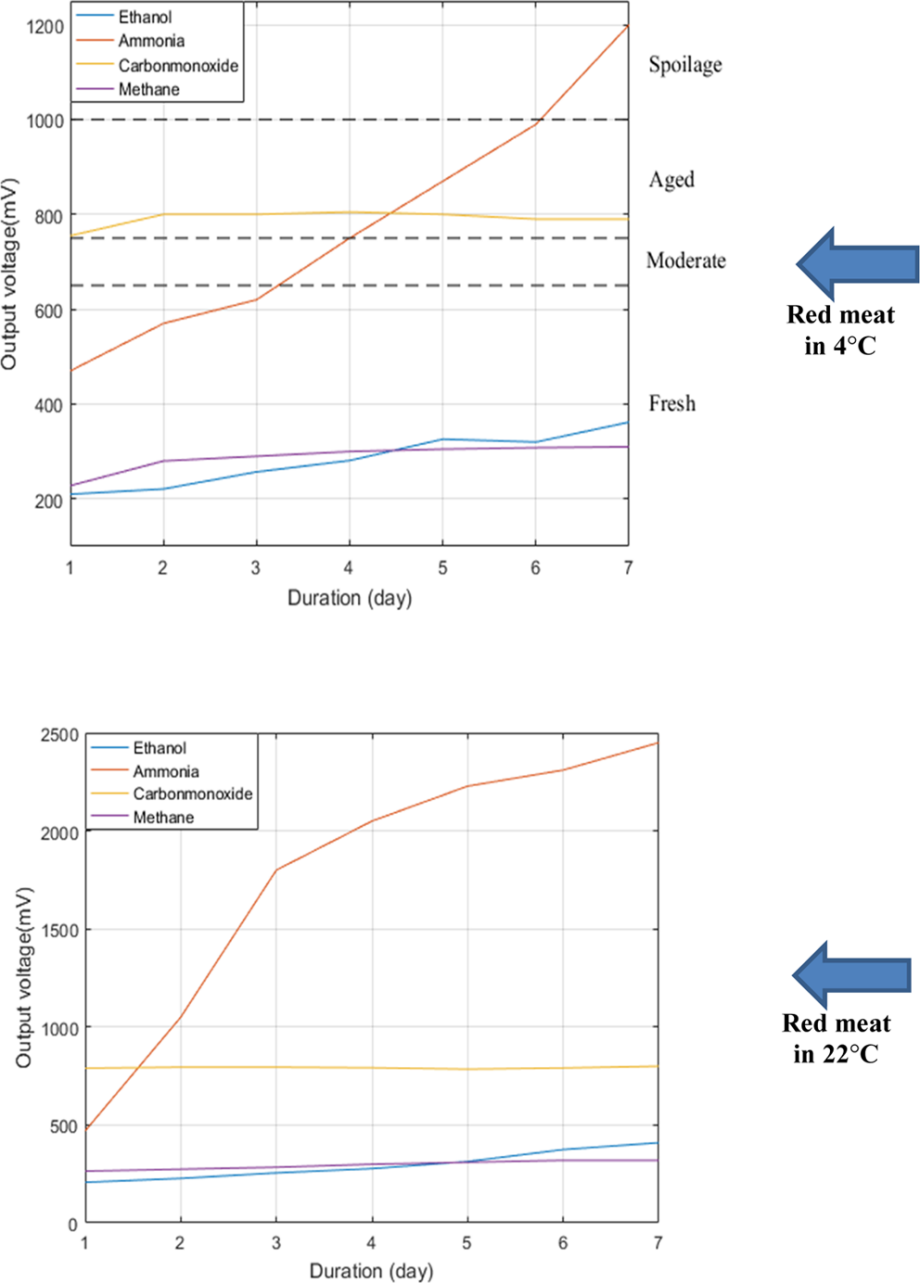
Concentration of ethanol, ammonia, carbon monoxide, and methane gases for red meat at (a) 4 °C and (b) 22 °C.

The degradation levels were not added to the graph in Figure 9 as well as in Figure 8 since the spoilage was observed only at the end of the first day according to the ammonia level while the changes in the other sensor values are negligible.

The critical values were established as 625, 725, and 1000 mV based on the ammonia curve from the study of Eom et al.^35^ because scaling would be more obvious and measurable among these sensor types ranging from 450 to 1200 mV with an increase rate of 166% approximately. According to the graph in Figure 9, freshness is maintained for up to 3 days in a refrigerator (+4 °C), while spoiling develops during a 6 day period. Food poisoning may develop if the age interval is between 630 and 730 mV. In summary, samples up to 730 mV are accepted as ready to consume, i.e., logic 0. Red meats, on the other hand, can be considered spoiled, starting with a value of 1000 mV (logic 1).

## 4 Discussion

It is believed that there is a clear relationship between the e-nose sensor output values and the other analytical methods. However, precisely analyzing the outcomes under these conditions necessitates extensive periods of observation. On the other hand, this approach proves that the correlation can be calculated easily according to the measured data. The desired sensory values are obtained as is expected regardless of considering GC-MS and microbial analysis. Furthermore, the total number of bacteria steadily grows between intervals, and GC-MS verifies this relationship by considerably contributing to the detection of these gases in the headspace sampling method.

Generally, meat is divided into red and white meat. Sheep, beef, and goat meat are red meat; the poultry meat and fish such as chicken and turkey are white meat. Proteins called myoglobin are what give meat its red color. The amount of myoglobin in red meat is much higher than that in white meat. After the meat is cut, various biochemical events and enzymatic reactions occur. Immediately afterward, if the meat is not stored under appropriate conditions, microorganisms become active and the process of loosening, softening, and deterioration begins in the meat over time.^36^

The threshold value of spoilage for red meat was determined according to the study by Eom et al.^35^ As they explained in their study, this spoilage threshold value (1000 mV) selected where the smell and color of poultry meat were very poor. The result showed quite a similar trend in this study compared to the study by Eom et al. Besides this, the slope of graph significantly increased at this point in Figure 8. Likewise, the other spoilage value for the poultry meat also shows the same slope increase in the transition point from aged to spoilage in Figure 9.

Comparing e-nose measurement results to GC-MS results, it can clearly be seen that this model is suitable for detecting harmful gases whether the meat freshness is adequate or not. Though the focus is on which compounds are dominant for the sample environment while GC-MS operation is selected half-manually, the existence of other more dominant gases in the unfocused zone does not affect the importance of this study. In this point of view, ethanol and ammonia are dominant gases among other compounds given in Figure 6 depending on the focus zone of selected molecular mass. In other words, the amount of ethanol and ammonia compounds in the environment of red and poultry meats using this e-nose design gives critical information about the spoilage level or freshness. Thus, this approach allows for the reliable usage of this design as decisive of meat freshness level.

Another important point to check the freshness or spoilage of meat is its smell. In the case of putrefaction, which occurs as a result of microorganism activities, meat becomes unusable. Generally, when kept in a hot environment, meat spoils and releases a disturbing, unpleasant odor, which can be easily felt in that environment.^37^ As a result of advanced oxidation, unpleasant greenish, yellowish, or very light meat colors occur. Meat color is one of the most obvious indicators of putrefaction and is extremely important not only for sensory purposes but also for consumer health.^38^ A slimy structure sometimes forms on the meat surface due to microbial activities. The meat becomes slippery due to the formation of microorganisms on its surface.^39^

During the putrefaction process of red meat with high myoglobin content, compounds such as ammonia and hydrogen sulfide are released as a result of the breakdown of amino acids, causing the meat to smell bad. During putrefaction, the meat takes on a color ranging from brown to green. If hygiene and sanitation are not followed during slaughter and after slaughter and if the meat is stored under inappropriate conditions, putrefaction accelerates due to microbial spoilage.^28,40^ Within the scope of this study, the amount of ammonia released as a result of microbial spoilage of red meat was measured by GC analysis, and it was observed that the amount of ammonia released due to microbial increase also increased.^29^

Poultry meat is easily perishable because it provides a suitable environment for the growth of microorganisms. The component that makes up the majority of the content of white meat (about 76%) is water. This consists of proteins with 20% and lipids with 3%. Other components found in small amounts in meat include carbohydrates, such as glucose, glucose-6-phosphate, glycogen, and various nitrogenous compounds. The first group of components used by microorganisms during the spoilage of poultry meat includes carbohydrate products such as glycogen, glucose-6-phosphate, and lactate. The second group includes metabolic products such as gluconate, gluconate-6-phosphate, and pyruvate. The last group consists of nitrogenous energy sources such as amino acids.^31^ The first component that spoilage microorganisms will prefer to use is glucose. Second, the preferred carbohydrate under aerobic and anaerobic conditions is lactate. In general, the last preferred component is amino acids. It is known that in poultry meat, unlike in the meat of other animal species, fat does not spread between muscle tissues, and most of it is located under the skin and in the abdominal cavity. This makes the work of microorganisms easier, and deterioration in poultry generally begins on the skin, the fat parts associated with the skin, and the outer surfaces of the muscles. In the logarithmic reproduction phase of spoilage microorganisms, short-chain fatty acids, ketones, and alcohols that do not cause bad odor are released as a result of glucose metabolism.^32,40^ The components that will create the bad odor are released when the number of microorganisms is >107/cm^2^, the glucose level decreases, and components such as lactate and amino acids are used.^41,42^ In light of this information, within the scope of this study, the amount of ethanol released as a result of microbial spoilage of poultry meat was measured by GC analysis, and it was observed that the amount of ethanol released due to the microbial increase on white meat increased.

Calibration is another enhancement issue that can be addressed to improve the measurement correlation. The main measurements were performed by sensors to create a reliable data processing system. As a result, the sensor values were predicted to be decisive in determining whether the samples are fit for consumption or not. In this context, given that one of the most important points is to calibrate the connected sensors, particularly gas sensors, they should be calibrated based on the type of conversion done in the software command. In this study, given that ppm measurements were not taken in this investigation, calibrations were performed by using the output voltages.

## 5 Conclusions

Traditional techniques, such as GC-MS analysis, will always be required in the near future to describe quantitatively or qualitatively the difference between other food product samples. Other measurements are only carried out to verify data gained from sensor outputs since these processes are time-consuming, expensive, and need preoperational effort, even if they provide vital data that would be required for other measurement techniques. Similarly, bacterial analysis is an important step in determining what type of bacterial groups are responsible for the gases detected by GC-MS analysis throughout the degradation process. In this regard, it may be strongly argued that increasing the number of higher sensors expands the applicable region. Although adding several separate sensors is expected to take up much more space, the total circuit area would not expand linearly in the same way since several output pins can connect mutually. This system can easily be made portable and takes up very little space. The main measurements are sensory in nature.

Sensory measurements show reliable and characteristic data compared to other studies and other verification methods in the study such as GC-MS and microbial analysis. Selecting threshold values for transition points was made not only for smell and color but also for slope increase in this point as much as possible. Since it is not considered quite significant whether averaging the data or single-shot measurement, the sensor output values in these points were not saved as multimeasurements.

There are several improvements that can be made to maximize the impact area of the system. As the number of studies in this field increases, the most portable, easy-to-apply, and fastest-performing systems will be developed in a similar way. Focusing and performing the developed methods frequently with minor differences will also increase the reliability of such systems and pave the way for their widespread use. Thus, even individual widespread use of similar methods will lower the price, and in the near future, these measurements could be compulsory for the suppliers. The general trend in this area is to build up a system for many specialized applications to measure freshness quality.

In light of all of these considerations, this approach suggests that portable and compact equipment or an experimental product would be ideal. Nonetheless, the study in this form is suitable to serve as a reference for future researchers in developing road maps with good repeatability and stability.
